# Idiopathic inflammatory myopathy associated with Sjögren’s disease: features of a distinct clinical entity

**DOI:** 10.3389/fimmu.2025.1654576

**Published:** 2025-09-09

**Authors:** Franz Felix Konen, Yunus Emre Güzeloglu, Tabea Seeliger, Konstantin Fritz Jendretzky, Sandra Nay, Lea Grote-Levi, Philipp Schwenkenbecher, Christine Gründges, Diana Ernst, Torsten Witte, Thomas Skripuletz

**Affiliations:** ^1^ Department of Neurology, Hannover Medical School, Hannover, Germany; ^2^ Department of Rheumatology and Clinical Immunology, Hannover Medical School, Hannover, Germany

**Keywords:** Sjögren´s disease, idiopathic inflammatory myositis, inclusion body myositis, polymyositis, dermatomyositis, overlap syndrome

## Abstract

**Background:**

Idiopathic inflammatory myopathies (IIM) and Sjögren’s disease (SjD) may coexist, but data on their combined presentation and treatment response remain limited.

**Methods:**

We retrospectively analyzed 23 patients with biopsy-confirmed IIM and coexisting SjD, compared to 24 age- and sex-matched IIM controls without SjD. Clinical, electrophysiological, and immunological data, as well as treatments and outcomes, were assessed. Outcome assessment included EULAR Sjögren’s Syndrome Disease Activity Index (ESSDAI) and the ACR/EULAR Myositis Response Criteria for adult polymyositis and dermatomyositis.

**Results:**

Among IIM-SjD patients, 39% had inclusion body myositis (IBM), 61% had poly- or dermatomyositis. Compared to controls, asymmetric muscle weakness (78%, p=0.0012), non-muscular manifestations (52%, p=0.0035), and more immunosuppressive therapies (median 3; p=0.0253), including more frequent anti-CD20 use (30%, p=0.0039) were found in IIM-SjD. After a median follow-up of 80 months, patients showed better outcomes (lower ESSDAI and higher ACR/EULAR response scores; p=0.0031 and p=0.0083). IBM was a strong predictor of higher ESSDAI scores at follow-up (p=0.014).

**Conclusions:**

The study suggests that IIM-SjD is characterized by more asymmetric muscle weakness and extramuscular involvement. Enhanced immunosuppression led to better outcomes in patients with poly- or dermatomyositis, while IBM was linked to higher disease activity. Further research is needed to clarify underlying mechanisms.

## 1 Introduction

Idiopathic inflammatory myopathies (IIM) are rare, immune-mediated muscle diseases marked by progressive skeletal muscle weakness and chronic inflammation (1, 2). The main subtypes, polymyositis, dermatomyositis and inclusion body myositis (IBM), present with varying clinical and immunopathological features (1, 2). Polymyositis and dermatomyositis often involve proximal muscle groups and respond to corticosteroids and immunosuppressants such as methotrexate. In contrast, IBM typically presents with asymmetric distal weakness in predominantly older individuals and shows poor response to conventional immunosuppression (1–4). IIM are myopathies of autoimmune origin, with both T and B cells contributing to tissue damage (1–4). Autoantibodies and aberrant B-cell activity, key components in dermatomyositis and polymyositis, support the use of targeted B-cell therapies such as rituximab (5, 6). In patients with IIM, other comorbid autoimmune diseases like Sjögren’s disease (SjD) can be found. SjD is a rare, systemic autoimmune disorder affecting exocrine glands, leading to the hallmark sicca symptoms (7, 8). Diagnosis relies on ACR/EULAR classification criteria incorporating serologic (anti-SSA/Ro), functional (Schirmer test, salivary flow), and histopathological parameters (9, 10). The choice of immunomodulatory treatment depends on the severity of clinical manifestations, ranging from local measures to systemic medications like hydroxychloroquine or rituximab (11–13). Beyond glandular involvement, extraglandular manifestations including neurological conditions are common and can be found in up to 50% of the patients (14). Neurological involvement in SjD can affect the peripheral and central nervous system as well as the muscles and thus lead to different neurological deficits (15–20). It is controversial discussed if SjD and IIM are only comorbidities or if SjD causes myositis. Retrospective and prospective studies suggest that among myositis subtypes, IBM is most frequently associated with SjD, often presenting with anti cN1A antibodies, asymmetric distal muscle weakness, and non-response to immunosuppression (21–30). In contrast, polymyositis and dermatomyositis are less commonly associated with SjD and, when present, typically manifest with classical proximal muscle involvement and a better treatment response. However, some studies report subclinical myositis in up to 72% of SjD patients without overt muscle symptoms (21–30). Patients with IIM-SjD are often younger at disease onset and more frequently require combination immunosuppressive therapy, yet do not necessarily exhibit higher SjD disease activity compared to those with isolated SjD (21–30). In the present study, patients with IIM and concomitant SjD were investigated and compared to IIM patients without SjD in order to identify differences and similarities in clinical presentation, electrophysiological findings, therapeutic strategies and disease course, thus further illustrating the reported associations.

## 2 Material and methods

### 2.1 Patients

Patients with muscle biopsy confirmed IIM presenting to Hannover Medical School between 2015 and 2024 were screened for coexisting SjD. Screening included Saxon- und Schirmer-testing as well as detection of SS-A antibodies in the first place and a glandular biopsy in antibody-negative cases with xerophthalmia and/or xerostomia. Salivary gland biopsy was also offered to antibody-positive cases, but not all of these patients consented to perform the biopsy since the diagnosis of SjD was already established applying the screening procedure only. Regardless of any prior SjD diagnosis established in outpatient or inpatient settings elsewhere, all patients underwent a systematic assessment for SjD at Hannover Medical School. SjD was diagnosed according to the 2016 ACR/EULAR classification criteria for primary SjD (9). If SjD was confirmed, the patient was included in the further analyses of this study. Clinical and paraclinical data were retrospectively analyzed with a special focus on diagnosis, involvement of IIM besides striated skeletal musculature, disease course and treatment. In addition to demographic data, results of electromyography and electroneurography, laboratory analyses, treatment details, patient acceptable symptom state (PASS), and clinical improvement were assessed (3, 31).

To assess disease activity in patients with SjD, the ESSDAI (EULAR Primary Sjögren’s Syndrome Disease Activity Index) and ESSPRI (EULAR Primary Sjögren’s Syndrome Patient-Reported Index) scores were calculated (32, 33). To evaluate clinical improvement in patients with IIM, the ACR/EULAR criteria for minimal, moderate, and major clinical response in adult dermatomyositis and polymyositis were applied (3, 34–36). Due to the retrospective nature of the study, only the minimal dataset comprising Manual Muscle Testing, Extra-muscular Disease Activity, and Muscle Enzymes was used (3, 34–36).

**Figure 1:**
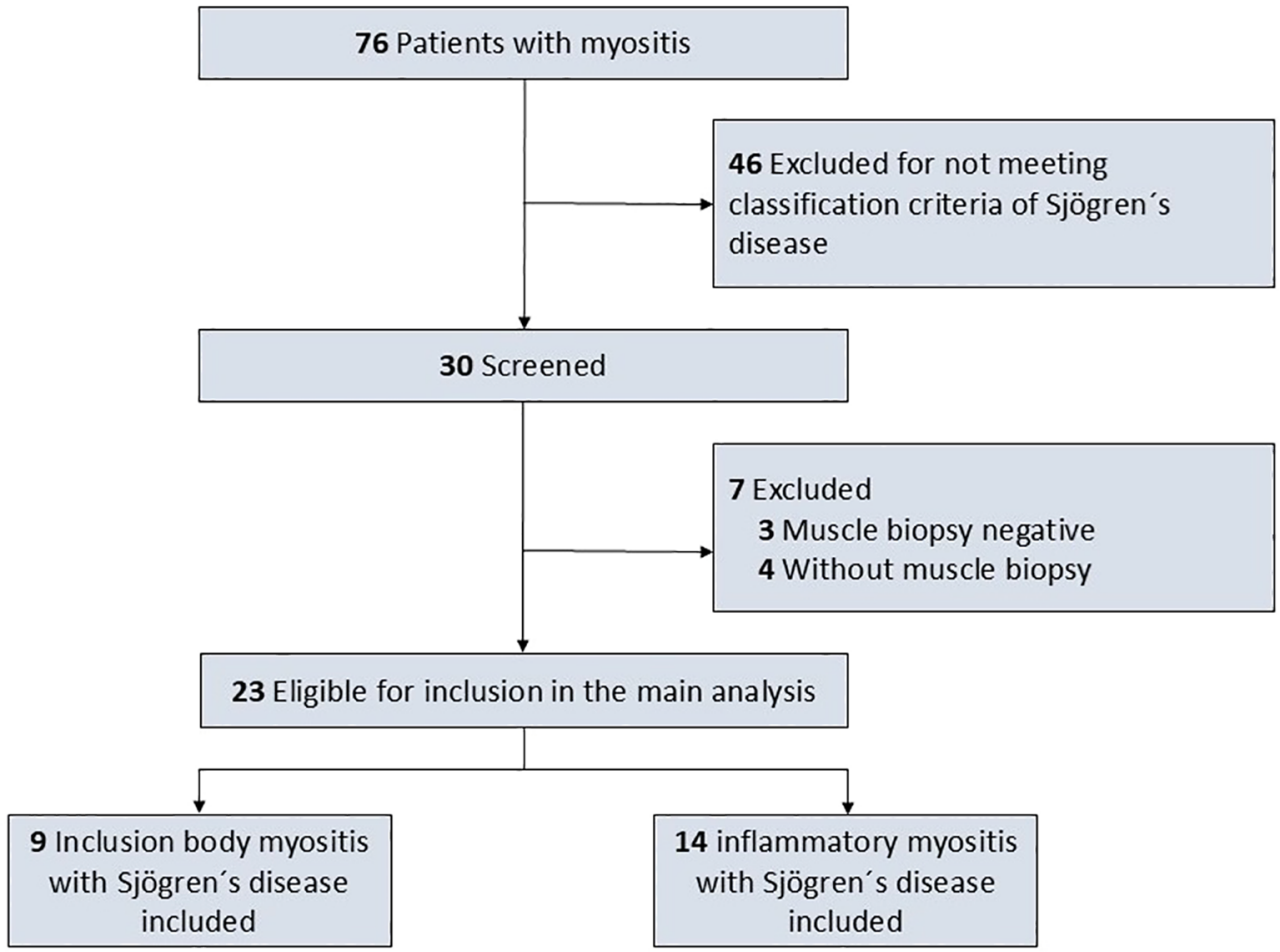
Study flowchart.

The findings were compared to sex- and age-matched controls with biopsy confirmed IIM in whom SjD was ruled out applying the 2016 ACR/EULAR classification criteria for primary SjD applying a frequency matching approach (9). To achieve a balanced matching for sex and age while ensuring exclusion of SjD, 13 patients with IBM and 11 patients with dermato-/polymyositis were included as controls. Patients and controls were excluded from the present study if the diagnosis of IIM was not confirmed by muscle biopsy. Histopathological results of muscle biopsy of the included patients was defining the clinical diagnosis of IBM, dermatomyositis and polymyositis, thus matching also accounted for histopathological findings. **Figure 1** depicts the flowchart of the study.

### 2.2 Statistics

All statistical analyses were performed using standard software (IBM SPSS Statistics, version 29.0, IBM Corp., Armonk, NY, USA). Continuous variables were described as median and interquartile range (IQR), and categorical variables as absolute and relative frequencies. A two-sided significance level of 5% (p < 0.05) was considered statistically significant for all comparisons. For variables with missing data, a complete-case analysis was performed; no imputation methods were applied. Prior to analysis, data were tested for normal distribution using the Shapiro-Wilk test. For normally distributed continuous variables, group comparisons were conducted using the unpaired (independent) t-test. In cases where the assumption of normality was violated, the non-parametric Mann–Whitney U test was applied instead. For categorical variables, group comparisons were conducted using the chi-square test or Fisher’s exact test where appropriate. For within-subject comparisons between baseline and follow-up, paired t-tests or Wilcoxon signed-rank tests were used depending on data distribution. To explore predictors of disease activity and treatment intensity, multivariate linear regression analyses were performed. Independent variables included demographic factors (SjD diagnosis, age at SjD diagnosis, age at IIM diagnosis, sex), clinical variables (IBM subtype, baseline ESSDAI, ESSPRI), immunological markers (anti-SSA/Ro and anti-SSB/La antibodies), and treatment-related variables (number of immunosuppressive agents, use of rituximab or cyclophosphamide). The dependent variables were ESSDAI score at last follow-up, delta-ESSDAI, manual muscle testing score at follow-up, delta muscle strength, and total improvement score. All models were tested for standard regression assumptions, including linearity, homoscedasticity, and normal distribution of residuals. Multicollinearity was assessed using variance inflation factors (VIFs), which did not indicate critical collinearity among predictors. In addition, an exploratory binary logistic regression model was applied to assess predictors of high-efficacy therapy use (rituximab, cyclophosphamide). Due to the small sample size and convergence issues, the results of this model are reported in the results section for exploratory purposes only but should be interpreted with caution.

## 3 Results

### 3.1 Patients

A total of 23 patients with SjD and concomitant IIM were included in the study. Of these, 39% (9 out of 23) were diagnosed with IBM, and 61% (14 out of 23) with either polymyositis (11 patients) or dermatomyositis (3 patients). At the time of SjD diagnosis, patients with IBM were significantly older than those with polymyositis or dermatomyositis (63 years versus 55 years, p=0.0089). In most cases, SjD was diagnosed concurrently with or after the diagnosis of IIM. Further details on SjD characteristics are provided in **Table 1**.

**Table 1:** Sjögren´s disease (SjD) in patients with idiopathic inflammatory myopathies (IIM).

Characteristic	Inclusion body myositis (n=9)	Poly- and dermatomyositis (n=14)	p-value for group comparison
Age at SjD diagnosis [years], median (IQR)	63 (60-75)	55 (45-59)	**0.0089**
Diagnosis of myositis			
before SjD diagnosis, n (%)	2 (22%)	3 (21%)	>0.9999
at SjD diagnosis, n (%)	1 (11%)	6 (43%)	0.1760
after SjD diagnosis, n (%)	6 (67%)	5 (36%)	0.2138
Objective xerophthalmia, n (%)	7 (78%)	9 (64%)	0.6570
Objective xerostomia, n (%)	4 (44%)	9 (64%)	0.4173
Anti-SSA/Ro-antibody positive, n (%)	7 (78%)*	12 (86%)*	>0.9999
Anti-SSB/La-antibody positive, n (%)	2 (22%)	6 (43%)	0.3998
Sialadenitis grade ≥ 3 (Chisholm and Mason)	2/4 (50%)	3/5 (60%)	>0.9999

SjD, Sjögren´s disease; IQR, interquartile range; n, number; *all patients were positive for Ro52-antibody.Significant p-values are written in bold text values.

### 3.2 Baseline characteristics in IIM with and without SjD

When comparing baseline characteristics of patients with IIM and concomitant SjD to those without SjD, asymmetrical muscle weakness was observed significantly more often in patients with SjD (**Table 2**). This was particularly evident in patients with polymyositis and dermatomyositis, where asymmetrical muscle weakness occurred in 11 out of 14 patients with SjD, compared to only 1 out of 11 patients without SjD (p=0.0172). Manifestations of IIM besides of the striated skeletal musculature (involving heart, lungs, skin, or joints) were also significantly more frequent in patients with concomitant SjD (**Table 2**).

**Table 2:** Baseline characteristics of patients with idiopathic inflammatory myopathies (IIM) with and without Sjögren´s disease (SjD).

Characteristic	IIM and SjD (n = 23)	IIM controls without SjD (n = 24)	p-value
Age at myositis diagnosis [years], median (IQR)	57 (45-62)	63 (53-71)	0.0653
Females, n (%)	16 (70%)	16 (67%)	>0.9999
Time between muscle symptom onset and diagnosis [months], median (IQR)	13 (2-36)	23 (12-38)	0.2008
Manifestations besides of the striated skeletal musculature*, n (%)	12 (52%)	5 (21%)	**0.0355**
Muscular weakness focused			
on upper limbs, n (%)	0	2 (8%)	0.4894
on lower limbs, n (%)	8 (35%)	3 (13%)	0.0933
on upper and lower limbs equally, n (%)	15 (65%)	19 (79%)	0.3412
proximally, n (%)	16 (70%)	9 (38%)	**0.0415**
distally, n (%)	3 (13%)	4 (17%)	0.9999
proximally and distally equally, n (%)	4 (17%)	11 (46%)	0.0599
asymmetrically, n (%)	18 (78%)	7 (29%)	**0.0012**
Bulbar and respiratory involvement such as dysphagia, dyspnea, and dysarthria, n (%)	15 (65%)	18 (75%)	0.5343
Myalgia, n (%)	20 (87%)	20 (83%)	>0.9999
Serum creatinine kinase concentration at diagnosis [U/l], median (IQR)	512 (366-1366)	433 (241-1117)	0.1129
Evidence of pathognomonic myositis antibodies, n (%)	5 (22%)	3 (13%)	0.4614
Polyneuropathy, n (%)	14 (61%)	16 (67%)	0.7661
focus on upper limbs, n (%)	2 (15%)	1 (6%)	0.6085
focus on lower limbs, n (%)	3 (21%)	9 (56%)	0.0933
focus on both limbs equally, n (%)	9 (64%)	6 (38%)	0.3587
axonal pattern in ENG, n (%)	11 (79%)**	15 (94%)**	0.3155
demyelinating pattern in ENG with fulfillment of current CIDP criteria, n (%)	3 (21%)**	0**	0.1092
Electromyography			
Pathological spontaneous activity, n (%)	23 (100%)	23 (96%)	>0.9999
Decreased amplitudes, n (%)	15 (65%)	8 (33%)	**0.0422**
Interference pattern dense/sparse, n (%)	10 (43%)	16 (67%)	0.1468
Polyphasia, n (%)	16 (70%)	20 (83%)	0.3177
Usage of high-efficacy therapies (rituximab, cyclophosphamide), n (%)	8 (35%)	0	**0.0016**

IIM, idiopathic inflammatory myopathy; SjD, Sjögren´s disease; n, number; ENG, electroneurography; CIDP, chronic inflammatory demyelinating polyneuropathy [reference No. 37]. *involvement of heart, lungs, skin, or joints; **ENG available in 14/23 patients with IIM and SjD and 16/24 patients with IIM and without SjD.Significant p-values are written in bold text values.

Further details on the comparison of IIM patients with and without SjD are presented in **Table 2** and show no other clear differences between the two groups. Non-neurological comorbidities including cardiovascular (e.g. arterial hypertension, heart failure), metabolical (e.g. obesity, glucose metabolism disorders) and hormonal diseases (thyroid gland insufficiency) were frequently observed in both cohorts. Electromyography and electroneurography revealed significant differences only in the prevalence of reduced amplitudes in electromyography (**Table 2**). Regarding laboratory analyses, myositis-specific antibodies were detected with similar frequencies in patients with concomitant SjD (PM-Scl-100 + SAE1 + PM-Scl75, PM-Scl100, SRP, MDA5 + SRP, Mi-2alpha + Mi2ß, each n=1) and those without coexisting SjD (SRP (n=2), NXP2 (n=1)). SSA-antibodies were found in a total of 17/47 patients (IIM with and without SjD), thus being prevalent in 36% in the present cohort. Testing for anti-cN1A antibodies was not performed in patients with IBM.

### 3.3 Treatment in IIM with and without SjD

As shown in **Table 2** and **Supplementary Table 1**, IIM-SjD patients received significantly more different therapeutics than IIM patients without SjD during the disease course (median follow-up duration with SjD: 68 months (IQR: 32-102); median follow-up duration without SjD: 92 months (IQR: 49-147); p=0.2638). High-efficacy therapies (rituximab, cyclophosphamide) were significantly more often used in IIM-SjD patients than controls (**Table 2**).

### 3.4 Outcome parameters in IIM with SjD

**Figure 2:**
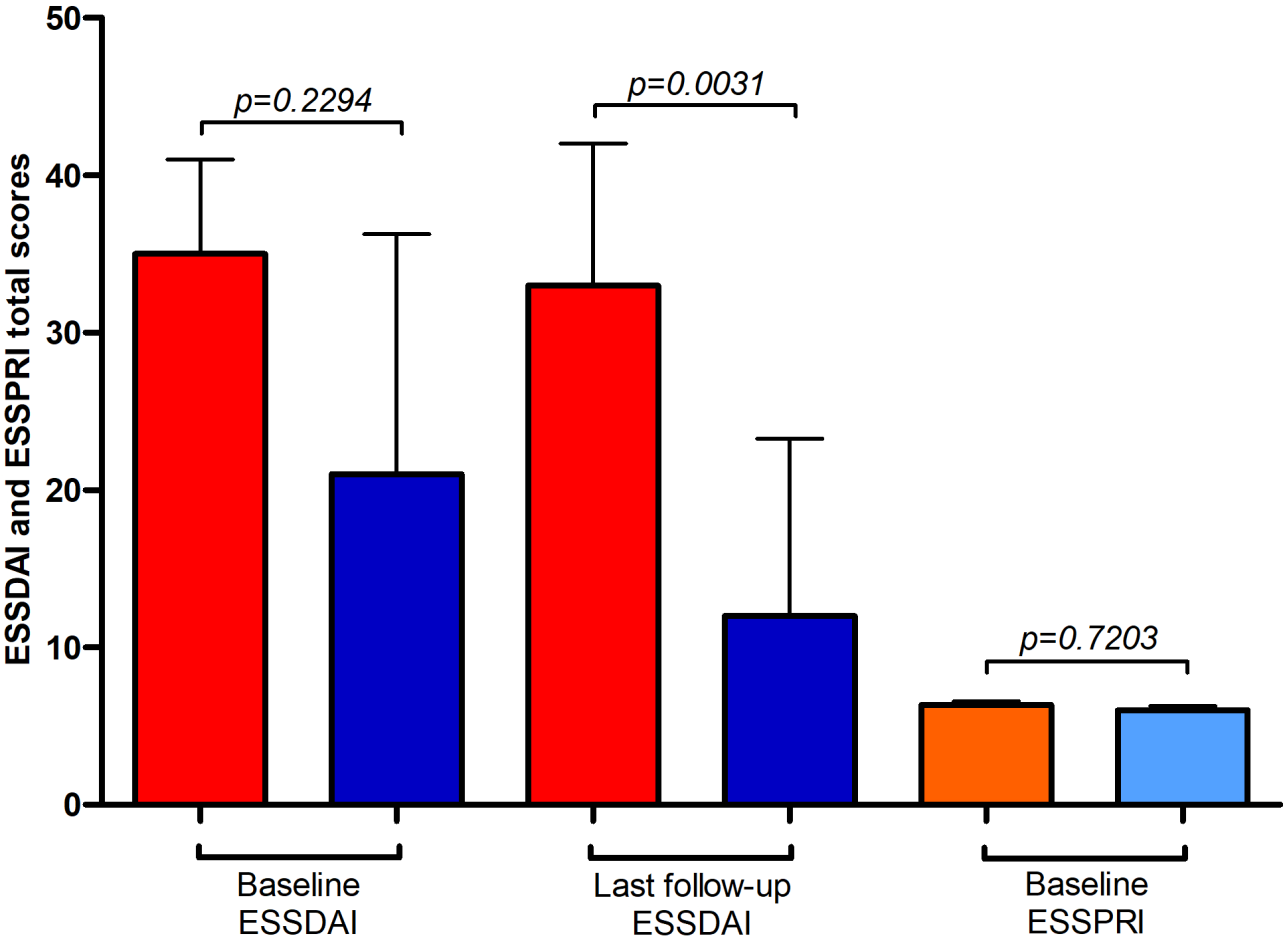
Outcome parameters in idiopathic inflammatory myopathy (IIM) with Sjögren´s disease (SjD). Red and orange columns represent patients with SjD and concomitant inclusion body myositis (IBM), whereas dark and light blue columns represent patients with SjD and concomitant polymyositis and dermatomyositis (PM, DM). Depicted are ESSDAI (EULAR primary Sjögren’s syndrome disease activity; red and dark blue columns) and ESSPRI (EULAR primary Sjögren’s syndrome patient-reported indices; orange and light blue columns) total scores at different time points. ESSPRI total score were available in n=9 patients each.

Median ESSPRI scores (IBM: 6.3, interquartile range (IQR): 3.7-6.5; polymyositis/dermatomyositis: 6, IQR: 4.3-6.5) as well as ESSDAI (IBM: 35, IQR: 25-41; polymyositis/dermatomyositis: 21, IQR: 17-36) at diagnosis were not significantly different comparing patients with IBM or poly-/dermatomyositis and comorbid SjD (**Figure 2**). At last follow-up (median of 68 months after diagnosis, IQR 32–102 months; IBM and SjD: 68 months (IQR: 36–144 months); poly-/dermatomyositis and SjD: 67 months (IQR: 24-93)), ESSDAI scores were significantly lower in poly-/dermatomyositis and comorbid SjD than IBM (IBM: 33, IQR: 28-42; polymyositis/dermatomyositis: 12, IQR: 11-23) as ESSDAI significantly improved in patients with polymyositis/dermatomyositis and SjD (p=0.0038), but did not change in patients with IBM (p=0.7518).

### 3.5 Outcome comparison in IIM with and without SjD

In terms of treatment response, manual muscle testing was not significantly different comparing median scores (and IQR) at diagnosis and last follow-up in IIM-SjD patients (**Table 3**; all patients: p=0.8559; IBM: 67 (58-73) vs. 65 (52-73), p=0.0749; polymyositis/dermatomyositis: 74 (70-77) vs. 78 (70-80), p=0.1226). In contrast, in IIM patients without SjD, manual muscle testing worsened significantly, mainly due to the worsening of the IBM patient group (all patients: p=0.0171; IBM: 69 (62-73) vs. 63 (35-71), p=0.0283; poly-/dermatomyositis: 72 (65-78) vs. 70 (67-75), p=0.3342).

**Table 3:** Outcome of patients with idiopathic inflammatory myopathies (IIM) with and without Sjögren´s disease (SjD).

Characteristic	IIM with SjD (n = 23)	IIM without SjD (n = 24)	p-value
Manual muscle testing at diagnosis [max. 80], median (IQR)	72 (67-75)	70 (63-75)	0.4684
Manual muscle testing at last follow-up [max. 80], median (IQR)	73 (64-80)	69 (47-71)	**0.0387**
Subjective response to treatment	17 (74%)	18 (75%)	>0.9999
PASS at last follow-up, n (%)	14 (61%)	12 (50%)	0.5612
Total improvement score at last follow-up, n (%)*	17.5 (7-33)	5 (2.5-7.5)	**0.0083**
minimal clinical improvement, n (%)*	8 (35%)	3 (13%)	0.1635
moderate clinical improvement, n (%)*	3 (13%)	1 (4%)	0.6078
major clinical improvement, n (%)*	0	0	0.9999
Serum creatinine kinase concentration at last follow-up [U/l], median (IQR)	68 (42-213)	217 (141-338)	**0.0030**

IIM, idiopathic inflammatory myopathy; SjD, Sjögren´s disease; n, number; *according to ACR/EULAR criteria for minimal, moderate, and major clinical response in adult dermatomyositis and polymyositis; PASS, patient acceptable symptom state. In the patient groups with and without SjD, all patients with inclusion body myositis, dermatomyositis and polymyositis were included.Significant p-values are written in bold text values.

Although only a small proportion of patients fulfilled the ACR/EULAR criteria for minimal and moderate clinical improvement, total improvement score was significantly higher in patients with SjD (**Table 3**), which was due to the SjD patients with polymyositis/dermatomyositis (median total improvement score with SjD: 30 (IQR: 15-39); median total improvement score without SjD: 6 (IQR: 2-21); p=0.0114).

PASS was achieved equally at last follow-up in patients with IIM with and without SjD (**Table 3**).

At last follow-up, serum creatine kinase (CK) concentration was significantly lower in SjD patients than patients without SjD, mainly due to IBM patients (median serum CK concentration (U/l) in patients with SjD: 43 (IQR: 28-178); median serum CK concentration (U/l) in patients without SjD: 218 (IQR: 130-495); p=0.0122). Further information on treatment and outcome parameters is shown in **Table 3**.

### 3.6 Correlations with parameters of SjD disease activity

To assess for correlations of baseline characteristics with outcome parameters in IIM-SjD patients, multivariate linear regression analyses were employed with the dependent variables being ESSDAI score at last follow-up, delta-ESSDAI, manual muscle testing score at follow-up, delta muscle strength, and total improvement score. Of these analyses, only prediction of ESSDAI-score at last follow-up was statistically significant (delta-ESSDAI: p=0.092, manual muscle testing score: p=0.391, delta muscle strength: p=0.956, total improvement score: p=0.213, ESSDAI-score at last follow-up: p=0.009, adjusted R²: 0.778), with evidence of IBM as type of IIM being the most relevant predictor (p=0.014) as shown in **Supplementary Table 2**.

To assess for predictors of usage of a higher number of immunosuppressants indicating a high disease activity, a multivariate linear regression analysis with number of immunosuppressants being the dependent variable was employed. The model was statistically significant (p=0.002, adjusted R²=0.831). As shown in **Supplementary Table 3**, higher baseline ESSDAI scores (p=0.021) and the presence of anti-SSB/La antibodies (p=0.025) were positively associated with the number of immunosuppressive agents used, whereas higher age at SjD diagnosis was negatively associated (p=0.011). In addition, an exploratory binary logistic regression was performed to assess predictors of high-efficacy therapy use. Although the overall model was statistically significant (χ²=21.270, p=0.006), convergence issues and extremely large standard errors indicated numerical instability, thus individual predictor estimates were not reported in detail.

## 4 Discussion

In this study, we examined patients with IIM and coexisting SjD, focusing on clinical presentation, treatment strategies, and outcomes. Our findings are largely consistent with previous reports describing the overlap syndrome of SjD and IIM, particularly IBM, and contribute important clinical observations.

The clinical presentation in our cohort closely resembled the features described in earlier studies of patients diagnosed with both IIM and SjD. Colafrancesco et al. and Felten et al. reported that patients often present with classical sicca symptoms alongside proximal muscle weakness and elevated muscle enzyme levels, with muscle biopsy findings most commonly indicating polymyositis or IBM (26, 28). Notably, our cohort included both IBM and polymyositis/dermatomyositis patients with SjD, reflecting the findings of Felten et al., where polymyositis and IBM were the most commonly observed subtypes associated with SjD (26). Consistent with observations by Chung et al. and Giannini et al., patients with IBM in our study tended to be older at diagnosis and frequently exhibited the characteristic asymmetric distal weakness and resistance to therapy (22, 25). Our data further support the notion that IBM may dominate the clinical phenotype in overlap cases and represent a key determinant of disease trajectory.

An important, yet underreported aspect of the combination of both diseases is the high prevalence of clinically apparent polyneuropathy. In our cohort, more than 60% of patients with both SjD and coexisting IIM exhibited polyneuropathic symptoms, including sensory deficits and reduced reflexes. However, in the present study, the prevalence of polyneuropathy was not significantly different comparing patients with and without SjD. Although most of these patients showed axonal damage patterns on electroneurography, up to 21% of patients with SjD (compared to none without SjD) presented a demyelinating damage pattern with fulfillment of the currently applied diagnostic criteria for chronic inflammatory demyelinating polyneuropathy (CIDP) (37). In contrast, none of the patients in the control group fulfilled the CIDP criteria (37). These findings are notable, as they expand on the work by Levy et al., who reported peripheral nervous system involvement in a significant number of patients with SjD and IBM overlap, suggesting that neurologic comorbidity may be an intrinsic feature of the overlap syndrome rather than a coincidental occurrence (24). While Chung et al. and Kanellopoulos et al. described occasional neuropathic symptoms in such cases, our study emphasizes this aspect with greater clarity and confirms the substantial burden of neurological symptoms in this patient population (25, 29). The concurrent involvement of muscle and nerve may indicate a broader immune-mediated neuromuscular process, as previously hypothesized by Nelke et al. (23).

Therapeutically, patients with additionally SjD received a significantly greater number of immunomodulatory treatments compared to patients with IIM alone, including a notably higher use of high-efficacy agents such as rituximab and cyclophosphamide. This observation is consistent with previous reports by Felten et al. and Giannini et al., who described more frequent and aggressive immunosuppressive regimens in overlap cases, often driven by systemic involvement and autoimmune B-cell activation (22, 26). Interestingly, the higher treatment burden in our patients with SjD was associated with significantly better clinical outcomes, as measured by the total improvement score, which reflects gains in muscle strength, normalization of muscle enzymes, and physician-assessed disease activity (34–36). This apparent paradox - more intensive therapy yielding better outcomes - may reflect a more personalized immunotherapy or possibly a greater treatment responsiveness in polymyositis or dermatomyositis overlap phenotypes. In contrast, IBM remains largely resistant to immunosuppressive treatment, as confirmed by multiple studies (25, 29). The more frequent use of rituximab in patients with SjD aligns with its targeted effect on B-cell dysfunction, a pathogenic feature common to both conditions (21, 27).

Furthermore, our multivariate analysis revealed that the presence of IBM was a strong predictor of higher ESSDAI scores at the last follow-up, indicating a persistently elevated systemic disease burden in this subgroup. This finding supports previous observations by Giannini et al. and Zeng et al., who reported that IBM in the context of SjD is associated with more refractory and multisystem involvement (21, 22). Additionally, the presence of anti-SSB/La antibodies emerged as a predictor for the use of a higher number of immunosuppressive agents, consistent with reports by Levy et al. and Rietveld et al., suggesting that anti-SSB/La-positive patients may display a broader autoimmune phenotype and are more likely to require intensified treatment (24, 27). In our cohort, anti-SSA antibodies were detected in 36% of all IIM patients, reflecting the relevance of systematic serological screening and underscoring the overlap between IIM and SjD on an immunological level. However, therapeutic decisions were not blinded in the clinical routine, thus treatment escalation might have been influenced by the clinical diagnosis of concomitant SjD. This may have introduced a degree of confounding by indication, which should be considered when generalizing associations between antibody status and treatment intensity. Given that anti-SSB/La antibodies are less commonly detected in isolated myositis, their predictive value in overlap syndromes warrants further prospective validation.

The present study is not free of limitations. The most important consisted in the limited sample size due the low prevalence of IIM itself and the even lower number of patients with SjD with concomitant IIM, which limited the statistical power to detect smaller differences. Thus, no separated analysis between patients with polymyositis and dermatomyositis was possible, which would be desirable, since important differences in pathophysiology, clinical presentation, and treatment response could be obscured. However, the approach of grouping polymyositis and dermatomyositis patients together as a single category is not unusual and regularly done in clinical studies. Further, optimal matching could not have been achieved due to cohort constraints, thus patients with SjD were younger than their matching counterparts. This is of importance since residual age differences may confound the interpretation of the data provided. Additionally, the exploratory binary logistic regression model assessing predictors of high-efficacy therapy use was limited by small sample size and convergence issues, resulting in numerically unstable estimates that should be interpreted with caution. Moreover, the unexpectedly high adjusted R² values observed in some regression models, despite the small sample size, raise the possibility of overfitting, which may have led to an overestimation of effect sizes and should be considered when interpreting these findings. The other important limitation of the study is the retrospective nature of the study. Thus, treatment decisions were not blinded introducing a potential confounding by indication. Selection bias cannot be ruled out, as patients with more complex disease courses and patients with SjD may have been more likely to be referred to our specialized tertiary center. Furthermore, the case-control design inherently limits causal inference and increases susceptibility to unmeasured confounders.

In conclusion, the combination of IIM and SjD constitutes a distinct clinical entity, frequently involving IBM and marked by systemic disease burden, neurological comorbidity, and intensified immunosuppressive treatment. Undergoing a higher treatment intensity, patients with polymyositis or dermatomyositis and SjD showed better outcomes, likely due to targeted treatment of the underlying SjD. However, prospective studies are needed to validate these findings and further elucidate causal relationships.

## Data Availability

The raw data supporting the conclusions of this article will be made available by the authors, without undue reservation.

## Supplementary material

Supplementary Table 1 Treatment of patients with idiopathic inflammatory myopathies (IIM) with and without Sjögren´s disease (SjD).

Supplementary Table 2 Results of multivariate regression analysis to predict ESSDAI at last follow-up.

Supplementary Table 3 Results of multivariate regression analysis to predict number of used immunosuppressants.
