# Supplementary material for: Idiopathic inflammatory myopathy associated with Sjögren’s disease: features of a distinct clinical entity
Source: Front Immunol. 2025 Sep 9;16:1654576. doi: 10.3389/fimmu.2025.1654576 (PMC12454105; doi:10.3389/fimmu.2025.1654576)
Supplement: Supplementary Table 1 — Treatment of patients with idiopathic inflammatory myopathies (IIM) with and without Sjögren´s disease (SjD). [file DataSheet1.docx]

# Supplemental tables

**Supplemental table 1**. Treatment of patients with idiopathic inflammatory myopathies (IIM) with and without Sjögren´s disease (SjD).

|  | **IIM and SjD (n = 23)** | **IIM without SjD (n = 24)** | **p-value** |
| --- | --- | --- | --- |
| Number of immunomodulatory therapies, median (IQR)* | 3 (1-4) | 1 (1-3) | **0.0253** |
| Hydroxychloroquine, n (%) | 4 (17%) | 0 (0%) | **0.0496** |
| Corticosteroids, n (%) | 4 (17%) | 5 (21%) | >0.9999 |
| Azathioprine, n (%) | 8 (35%) | 10 (42%) | 0.7661 |
| Methotrexate, n (%) | 8 (35%) | 6 (25%) | 0.5343 |
| Mycophenolate mofetil, n (%) | 9 (39%) | 1 (4%) | **0.0044** |
| Ciclosporin, tacrolimus, sirolimus n (%) | 3 (13%) | 0 (0%) | 0.1092 |
| Intravenous/subcutaneous immunogloblins, n (%) | 18 (78%) | 17 (71%) | >0.9999 |
| Rituximab, n (%) | 7 (30%) | 0 (0%) | **0.0039** |
| Cyclophosphamide, n (%) | 1 (4%) | 0 (0%) | 0.4894 |

IIM = idiopathic inflammatory myopathy; SJD = Sjögren´s disease; n = number; * = without consideration of oral corticosteroids.

**Supplemental Table 2**. Results of multivariate regression analysis to predict ESSDAI at last follow-up.

| **Predictor** | **Standard error** | **Standardized β coefficient** | **95 %-CI** | **p-value** |
| --- | --- | --- | --- | --- |
| ESSDAI at diagnosis | 0.295 | 0.389 | [–0.183 – 1.161] | 0.141 |
| ESSPRI at diagnosis | 1.464 | –0.103 | [–3.961 – 2.591] | 0.654 |
| Anti-SSA/Ro-antibody positive | 6.875 | 0.251 | [–7.095 – 22.893] | 0.288 |
| Anti-SSB/La-antibody positive | 7.605 | 0.025 | [–15.890 – 17.292] | 0.929 |
| Age at SjD diagnosis [years] | 0.667 | 0.496 | [–0.981 – 2.081] | 0.437 |
| Age at IIM diagnosis [years] | 0.438 | –0.048 | [–1.071 – 0.952] | 0.897 |
| IIM subtype (IBM or PM/DM) | 4.863 | 0.597 | [4.756 – 26.718] | **0.014** |
| Sex | 7.615 | 0.043 | [–15.328 – 17.714] | 0.880 |
| Total number of immunosuppressants | 2.618 | 0.450 | [–2.853 – 8.663] | 0.304 |
| Usage of high-efficacy therapies (rituximab, cyclophosphamide) | 5.634 | 0.014 | [–11.959 – 12.775] | 0.944 |

ESSDAI = EULAR primary Sjögren's syndrome disease activity; ESSPRI = EULAR primary Sjögren's syndrome patient-reported indices; IIM = idiopathic inflammatory myopathy; SjD = Sjögren´s disease; IBM = inclusion body myositis; PM = polymyositis; DM = dermatomyositis.

**Supplemental Table 3**. Results of multivariate regression analysis to predict number of used immunosuppressants.

| **Predictor** | **Standard error** | **Standardized β coefficient** | **95 %-CI** | **p-value** |
| --- | --- | --- | --- | --- |
| ESSDAI at diagnosis | 0.028 | 0.411 | [0.015 – 0.145] | **0.021** |
| ESSPRI at diagnosis | 0.165 | 0.296 | [–0.059 – 0.673] | 0.101 |
| Anti-SSA/Ro-antibody positive | 0.788 | –0.285 | [–3.170 – 0.394] | 0.116 |
| Anti-SSB/La-antibody positive | 0.737 | 0.471 | [0.371 – 3.677] | **0.025** |
| Age at SjD diagnosis [years] | 0.058 | –1.128 | [–0.330 – –0.058] | **0.011** |
| Age at IIM diagnosis [years] | 0.058 | 0.142 | [–0.104 – 0.158] | 0.658 |
| IIM subtype (IBM or PM/DM) | 0.634 | –0.118 | [–1.911 – 0.951] | 0.471 |
| Sex | 0.879 | –0.351 | [–3.491 – 0.473] | 0.124 |
| Usage of high-efficacy therapies (rituximab, cyclophosphamide) | 0.685 | 0.207 | [–0.664 – 2.540] | 0.208 |

ESSDAI = EULAR primary Sjögren's syndrome disease activity; ESSPRI = EULAR primary Sjögren's syndrome patient-reported indices; IIM = idiopathic inflammatory myopathy; SjD = Sjögren´s disease; IBM = inclusion body myositis; PM = polymyositis; DM = dermatomyositis.
